# Supplementary material for: Identification, Characterization, and Functional Analysis of Chitin Synthase Genes in Glyphodes pyloalis Walker (Lepidoptera: Pyralidae)
Source: Int J Mol Sci. 2020 Jun 30;21(13):4656. doi: 10.3390/ijms21134656 (PMC7370082; doi:10.3390/ijms21134656)
Supplement: Supplementary file 1 [file ijms-21-04656-s001.pdf]

Table S1. The detail information of homologous genes of *GpCHSA* and *GpCHSB* in different species in phylogenetic analysis

Gene name	GenBank No.	Species	Phylogenetic group
OfCHSA	ACF53745.1	*Ostrinia furnacalis*	Lepidoptera
CmCHSA	AJG44538.1	*Cnaphalocrocis medinalis*	Lepidoptera
SeCHSA	AAZ03545.1	*Spodoptera exigua*	Lepidoptera
HaCHSA	QEQ50332.1	*Helicoverpa armigera*	Lepidoptera
CfCHSA	ACD84882.1	*Choristoneura fumiferana*	Lepidoptera
PxyCHSA	API6 1827.1	*Plutella xylostella*	Lepidoptera
HvCHSA	AZQ19982.1	*Heortia vitessoides*	Lepidoptera
BmCHSA	AFB83705.1	*Bombyx mori*	Lepidoptera
DmCHSA	NP_524233.1	*Drosophila melanogaster*	Diptera
MsCHSA	AAL38051.2	*Manduca sexta*	Lepidoptera
TcCHSA	AAQ55060.1	*Tribolium castaneum*	Coleoptera
LmCHSA	ACY38589.1	*Locusta migratoria*	Acrididae
AgCHSA	XP_321336.5	*Anopheles gambiae*	Culicidae
NlCHSA	XP_022187224.1	*Nilaparvata lugens*	Hemiptera
MbCHSA	ABX56676.2	*Mamestra brassicae*	Lepidoptera
SlCHSA	XP_022820393.1	*Spodoptera litura*	Lepidoptera
AmCHSA	XP_016770736.1	*Apis mellifera*	Hymenoptera
CmCHSB	AJG44539.1	*Cnaphalocrocis medinalis*	Lepidoptera
OfCHSB	ABB97082.1	*Ostrinia furnacalis*	Lepidoptera
HvCHSB	AZQ19981.1	*Heortia vitessoides*	Lepidoptera
HaCHSB	AKZ08595.1	*Helicoverpa armigera*	Lepidoptera
MsCHSB	AAX20091.1	*Manduca sexta*	Lepidoptera
SeCHSB	ABI96087.1	*Spodoptera exigua*	Lepidoptera
DmCHSB	NP_524209.3	*Drosophila melanogaster*	Diptera
TcCHSB	AAQ55061.1	*Tribolium castaneum*	Coleoptera
LmCHSB	AFK08615.1	*Locusta migratoria*	Acrididae
SfCHSB	AAS12599.1	*Spodoptera frugiperda*	Noctuidae
AgCHSB	XP_321951.2	*Anopheles gambiae*	Culicidae
BmCHSB	>AFC69002.1	*Bombyx mori*	Lepidoptera
PtCHSB	>XP_021004609.1	*Parasteatoda tepidariorum*	Araneae
AmCHSB	XP_001121152.2	*Apis mellifera*	Hymenoptera

Table S2 Primers used in RT-qPCR

Gene Names	Forward Primer (5'-3')	Reverse Primer (5'-3')
GpCHSA	TACGCTTTCCACATCACCGC	ACGGGCCTTCTCTTCCTTGT
GpCHSB	ACTTGGCTTTGGGCAGCTTT	GGTCCCTCGTCAACGCATTT
GpCDA1	TTCAAGCCATTCGCTGTCCC	CCAGGAAGCCATCTTGGCAG
GpCDA5	GTGCTTCCCTCCTAACACGC	CCCTTTCGATGGCAGGGTTC
GpCDA2	TTGGTGTGCGTGCTCCTTAC	TATGGGCGTTACCGTTGCAC
GpCHT-h	GCGACCCTTACAGAGGCAAC	TTTTCGCTTCACCGCATCGT
GpCHT3a	TTCAACGACTACAGCCCCGA	GAGAAGTAGCCGTTCAGGCG
GpCHT3b	TCGGGGAGAAGGTCGAGAGA	ATTCCAGGGCCAGTCGCAT
GpRpl32	CGATCACCTTCCGCTTCT	TGCTACCCAATGGCTTCC

Table S3 Primers used to synthesize dsRNA

Primer Names	Sequence(5′-3′)
GpCHSA-1-Olig-1	GATCACTAATACGACTCACTATAGGGGCTTACTACGTCATGCTTATT
GpCHSA-1-Olig-2	AATAAGCATGACGTAGTAAGCCCCTATAGTGAGTCGTATTAGTGATC
GpCHSA-1-Olig-3	AAGCTTACTACGTCATGCTTACCCTATAGTGAGTCGTATTAGTGATC
GpCHSA-1-Olig-4	GATCACTAATACGACTCACTATAGGGTAAGCATGACGTAGTAAGCTT
GpCHSA-2-Olig-1	GATCACTAATACGACTCACTATAGGGCCATATATCGCTTACCAAATT
GpCHSA-2-Olig-2	AATTTGGTAAGCGATATATGGCCCTATAGTGAGTCGTATTAGTGATC
GpCHSA-2-Olig-3	AACCATATATCGCTTACCAAACCCTATAGTGAGTCGTATTAGTGATC
GpCHSA-2-Olig-4	GATCACTAATACGACTCACTATAGGGTTTGGTAAGCGATATATGGTT
GpCHSB-1-Olig-1	GATCACTAATACGACTCACTATAGGGGCACCAGAACTCGGCATTTTT
GpCHSB-1-Olig-2	AAAAATGCCGAGTTCTGGTGCCCCTATAGTGAGTCGTATTAGTGATC
GpCHSB-1-Olig-3	AAGCACCAGAACTCGGCATTTCCCTATAGTGAGTCGTATTAGTGATC
GpCHSB-1-Olig-4	GATCACTAATACGACTCACTATAGGGAAATGCCGAGTTCTGGTGCTT
GpCHSB-2-Olig-1	GATCACTAATACGACTCACTATAGGGGCGAGGCATTACGTGCAATTT
GpCHSB-2-Olig-2	AAATTGCACGTAATGCCTCGCCCCTATAGTGAGTCGTATTAGTGATC
GpCHSB-2-Olig-3	AAGCGAGGCATTACGTGCAATCCCTATAGTGAGTCGTATTAGTGATC
GpCHSB-2-Olig-4	GATCACTAATACGACTCACTATAGGGATTGCACGTAATGCCTCGCTT
GFP-Olig-1	GATCACTAATACGACTCACTATAGGGGGGATGTCTCACATCTTGTTT
GFP-Olig-2	AAACAAGATGTGAGACATCCCCCCTATAGTGAGTCGTATTAGTGATC
GFP-Olig-3	AAGGGATGTCTCACATCTTGTCCCTATAGTGAGTCGTATTAGTGATC
GFP-Olig-4	GATCACTAATACGACTCACTATAGGGACAAGATGTGAGACATCCCTT

A

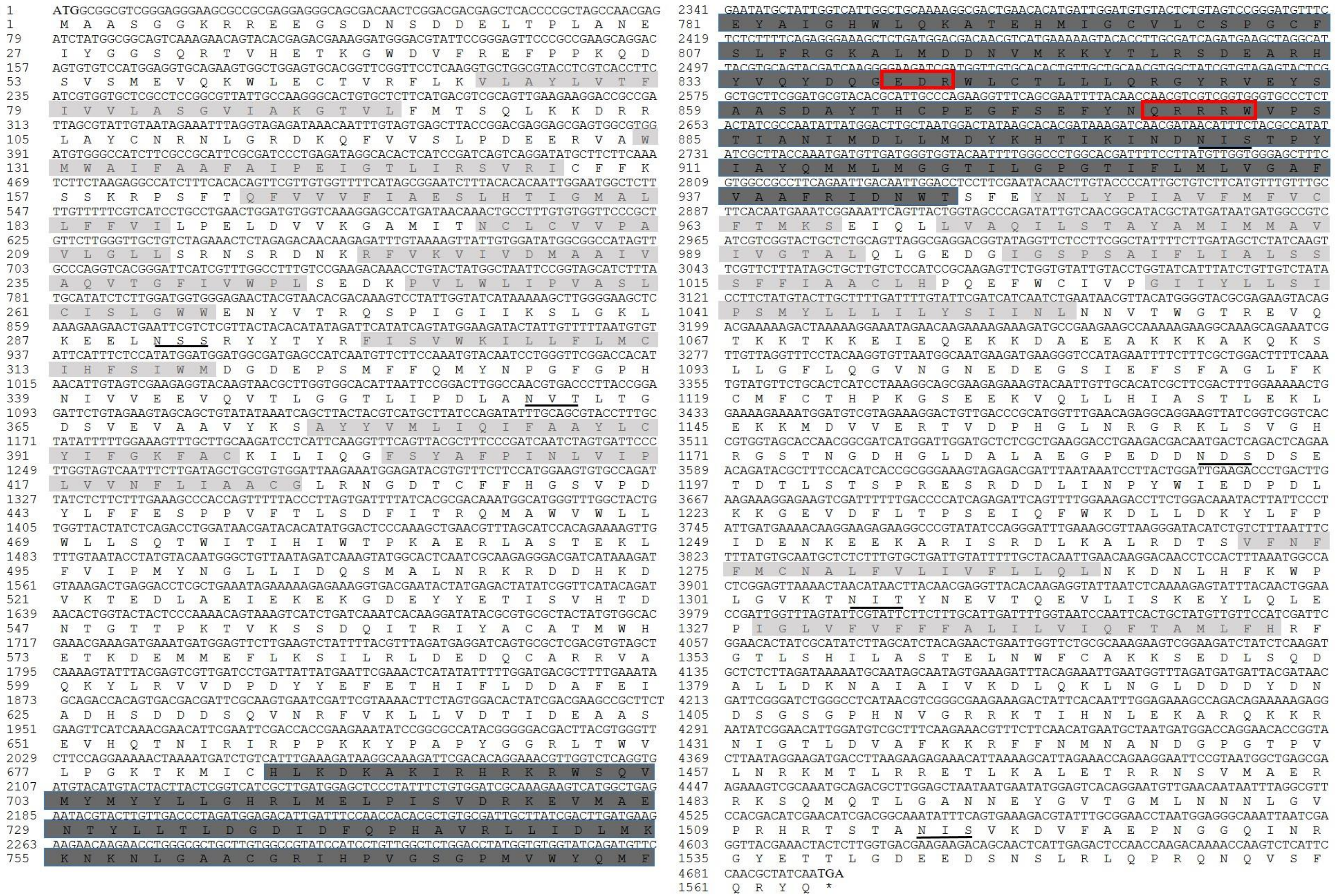

B

```
1     ATGACTTCAAATTCTGCTGGATTAAAGAAACCTTTGGAAGACAGTGATGATTCGGAATCGGATCTCACGCTACTGTAC
1      M  T  S  N  S  A  G  L  K  K  P  L  E  D  S  D  D  S  E  S  D  L  T  L  L  Y
79    GACGACTTAGACGAACCAGATCAAAGAACAGTCCAAGAAACGAAAGGATGGAACCTGTTCAGAGAGCTTCCGGTAAAG
27     D  D  L  D  E  P  D  Q  R  T  V  Q  E  T  K  G  W  N  L  F  R  E  L  P  V  K
157   AAGGAGAGCGGTTCGATGACATCGACCGCGTGGGTGGATACCATTCTGAAGATCATGAAGCTGCTGGCATACGTAGTC
53     K  E  S  G  S  M  T  S  T  A  W  V  D  T  I  L  K  I  M  K  L  L  A  Y  V  V
235   ATATTTGCATTAGTACTAGGCTCCGCAGTAGTTGCGAAAGGCACACTACTGTTTGTCTCATCACAACTCAAAAAAGGG
79     I  F  A  L  V  L  G  S  A  V  V  A  K  G  T  L  L  F  V  S  S  Q  L  K  K  G
313   AGACAAGTTGTTCTGTGTAATAGAGTAATAGCACCTGACAAGCAGTTCATAACAGTTCATACCTTCCATGAGCGCATA
105    R  Q  V  V  L  C  N  R  V  I  A  P  D  K  Q  F  I  T  V  H  T  F  H  E  R  I
391   ACTTGGCTTTGGGCAGCTTTTATAATATTTTCGGCACCAGAACTCGGCATTTGTATGAGATCCTTTAAAATATGTTTC
131    T  W  L  W  A  A  F  I  I  F  S  A  P  E  L  G  I  C  M  R  S  F  K  I  C  F
469   TTCAAAACAGTCAAGCAGCCCACCACTAAACAGTTTTTAGGGGCCTTCATCATAGAAACATGTCAAGCTGTAGGGATA
157    F  K  T  V  K  Q  P  T  T  K  Q  F  L  G  A  F  I  I  E  T  C  Q  A  V  G  I
547   GCTTTGTTGATTCTTTTCATCTTACCAGAGCTAGACGTAGTGAAAGGGGCTATGTTAATGAATGCACTTTGCTTCGTT
183    A  L  L  I  L  F  I  L  P  E  L  D  V  V  K  G  A  M  L  M  N  A  L  C  F  V
625   CCCGGGGTCCTAAATGCGTTGACGAGGGACCGTATGAATCCCAGATTCCCGTTGATGTTAATTTTGGACATTCTTGCT
209    P  G  V  L  N  A  L  T  R  D  R  M  N  P  R  F  P  L  M  L  I  L  D  I  L  A
703   GTATCTGCCCAAGCGACGGCCTTTGTCGTCTGGCCTCTGTTACAAGGAGAACCGATTCTTTGGACCATACCTGTAGCG
235    V  S  A  Q  A  T  A  F  V  V  W  P  L  L  Q  G  E  P  I  L  W  T  I  P  V  A
781   ACCATCCTTATATCGCTGGGTTGGTGGGAAAACTTTATAGGAAACGGCGACAAAAACACTCCAGGAATCGTACTAGCT
261    T  I  L  I  S  L  G  W  W  E  N  F  I  G  N  G  D  K  N  T  P  G  I  V  L  A
859   ATTATCACATTCAGAAAAGAACTTAAAACCAAGCGATACTATATATCAAGATATTTAAGTTTATGGAAAATATTTGTG
287    I  I  T  F  R  K  E  L  K  T  K  R  Y  Y  I  S  R  Y  L  S  L  W  K  I  F  V
937   TTTATGATATGCATATTAGCTTCTTTGTACGTCCAAGGAGATGATCCGTTAAGCTTCTTCACCCTAACAACAGAGGCA
313    F  M  I  C  I  L  A  S  L  Y  V  Q  G  D  D  P  L  S  F  F  T  L  T  T  E  A
1015  TTTAATGACCGAACATACACAGCTTATGAGGTACAAACTACATTACAGGATTTAGCAAACGGAATATATGAGTATGAG
339    F  N  D  R  T  Y  T  A  Y  E  V  Q  T  T  L  Q  D  L  A  N  G  I  Y  E  Y  E
1093  GTGACTGGCGATATAATTCCCGGTCTACCCATAGTTTGGACATCATCTCTGTGGGTGGCCCTAATCCAAGTCGGGATG
365    V  T  G  D  I  I  P  G  L  P  I  V  W  T  S  S  L  W  V  A  L  I  Q  V  G  M
1171  GCATACGTTTGTTACGTTAGCGGAAAACTGGCGTGCAAAATTCTGATACAGAACTTCAGTTACACTTTTGCATTGACT
391    A  Y  V  C  Y  V  S  G  K  L  A  C  K  I  L  I  Q  N  F  S  Y  T  F  A  L  T
1249  CTTGTAGGGCCAATGACTGTTAACCTATTGATTGTGTTTTCCGGATTGAGACATGAAAACCCGTGTGTATTTCATGGT
417    L  V  G  P  M  T  V  N  L  L  I  V  F  S  G  L  R  H  E  N  P  C  V  F  H  G
1327  ACCATACCGAATTATTTGTTCTTCGAAATGCCACAAGTGCAGAAACTAATGGACTTCGTGGAACGAGAGATGGCGTGG
443    T  I  P  N  Y  L  F  F  E  M  P  Q  V  Q  K  L  M  D  F  V  E  R  E  M  A  W
1405  GCTTGGCTCCTGTGGTTGGTGTCGCAGGCGTGGGTGTGCGTCCACGCGTGGATGCCGCGCTGCGAGAGGCTCGCTTCC
469    A  W  L  L  W  L  V  S  Q  A  W  V  C  V  H  A  W  M  P  R  C  E  R  L  A  S
1483  TCAGAGAAGCTCTTCGCCAAGCCGTGGTACTGTGGCCCGCTGATCGACCAGTGCCTCCTCATGAACAGGACGAAAGAC
495    S  E  K  L  F  A  K  P  W  Y  C  G  P  L  I  D  Q  C  L  L  M  N  R  T  K  D
1561  AATGCGATCGATGTTCCGATTGAGGACAAAGATCAGGAATATGATAATATATCGATCAATAGCGTGGATAAAGAAACA
521    N  A  I  D  V  P  I  E  D  K  D  Q  E  Y  D  N  I  S  I  N  S  V  D  K  E  T
1639  GACGTCAGATCCTCCGATCACACATCAAGAATATACATTTGTGCGACTATGTGGCACGAAACAAAAGAAGAAATGATA
547    D  V  R  S  S  D  H  T  S  R  I  Y  I  C  A  T  M  W  H  E  T  K  E  E  M  I
1717  GATTTCTTAAAGTCTATGTTCCGTCTCGATGAAGATCAGAGCGCGCGTCGCGTCGCTCAGAAGTACTGGGGCATCGTG
573    D  F  L  K  S  M  F  R  L  D  E  D  Q  S  A  R  R  V  A  Q  K  Y  W  G  I  V
1795  GATCCGGACTACTATGAATTAGAAGTTCACATGTTCATGGACGATGCATTCGAAATATCGGACCACAGCGCAGAAGAC
599    D  P  D  Y  Y  E  L  E  V  H  M  F  M  D  D  A  F  E  I  S  D  H  S  A  E  D
1873  TCGCAAGTGAATCGTTTCGTTAAGTGCCTAGTAGACACAGTGGATGAAGCAGCGTCTGAGGTCCATCTCACCAACGTG
625    S  Q  V  N  R  F  V  K  C  L  V  D  T  V  D  E  A  A  S  E  V  H  L  T  N  V
1951  AGGCTTCGGCCGCCCAAGAAATACCCCACGCCGTACGGCGGCCGGCTGGTGTGGACGATGCCCGGGAAGAACAAGATG
651    R  L  R  P  P  K  K  Y  P  T  P  Y  G  G  R  L  V  W  T  M  P  G  K  N  K  M
2029  ATCTGTCATCTAAAAGACAAGTCTAAGATCAGGCATAGGAAACGTTGGTCTCAGGTGATGTACATGTACTACTTCCTC
677    I  C  H  L  K  D  K  S  K  I  R  H  R  K  R  W  S  Q  V  M  Y  M  Y  Y  F  L
2107  GGTCACCGTCTGATGGACCTGCCGTTGTCGGTCGACCGTAAGGAAGTGATCGCCGAAAACACCTATCTTCTAGCCCTC
703    G  H  R  L  M  D  L  P  L  S  V  D  R  K  E  V  I  A  E  N  T  Y  L  L  A  L
2185  GACGGGGACGTCGATTTCAAGCCCGTCGCTGTCACTGTGCTTATCGATCTCATGAAGAAGAACAAGAACTTGGGAGCT
729    D  G  D  V  D  F  K  P  V  A  V  T  V  L  I  D  L  M  K  K  N  K  N  L  G  A
2263  GCTTGCGGGCGCATTCATCCGGTCGGATCTGGGCTACTGTCATGGTACCAGATGTTTGAGTACGCAATCGGTCATTGG
755    A  C  G  R  I  H  P  V  G  S  G  L  L  S  W  Y  Q  M  F  E  Y  A  I  G  H  W

2341  ATGCAGAAGGCGACTGAGCACATGATCGGATGCGTGCTCTGCAGCCCCGGCTGCTTCTCGCTGTTCAGAGGAAAGGCT
781    M  Q  K  A  T  E  H  M  I  G  C  V  L  C  S  P  G  C  F  S  L  F  R  G  K  A
2419  CTCATGGACGACAACATCATGAAGAAGTACACGCTCACGGCCAGTGAAGCGAGGCATTACGTGCAATACGATCAAGGG
807    L  M  D  D  N  I  M  K  K  Y  T  L  T  A  S  E  A  R  H  Y  V  Q  Y  D  Q  G
2497  GAAGACCGTTGGTTGTGTACTCTGCTGCTGCAGCGCGGCTACCGAGTCGAGTATTCAGCCGCCTCCGACGCGTACACG
833    E  D  R  W  L  C  T  L  L  L  Q  R  G  Y  R  V  E  Y  S  A  A  S  D  A  Y  T
2575  CATTGCCCCGAGGCCTTCAATGAATTCTACAACCAGCGGCGCCGCTGGGTGCCCTCCACCATGGCCAACATCCTTGAC
859    H  C  P  E  A  F  N  E  F  Y  N  Q  R  R  R  W  V  P  S  T  M  A  N  I  L  D
2653  TTGCTGACAGACTCTAAACGAACCGTTAAAATCAACGACAATATATCTACACTTTACATCATGTACCAGATCATGTTG
885    L  L  T  D  S  K  R  T  V  K  I  N  D  N  I  S  T  L  Y  I  M  Y  Q  I  M  L
2731  ATGGTGGGCGCCGTCCTAGGCCCGGGCACCATCTTCCTGATGATGATAGGTTCCATCAACGCTACCACCGGCCTGAGT
911    M  V  G  A  V  L  G  P  G  T  I  F  L  M  M  I  G  S  I  N  A  T  T  G  L  S
2809  AACATGAACGCGATGTTCGTGAACCTCGTACCAATAACTATCTACATTATAGTCTGTATGACCTGCAAAACTGAGATT
937    N  M  N  A  M  F  V  N  L  V  P  I  T  I  Y  I  I  V  C  M  T  C  K  T  E  I
2887  CAGCTGATTCTGGCAAACCTAATAACGTGCGCTTACGCCATGGTTATGATGATGGTGGTGATCGGCATTATGCTCCAA
963    Q  L  I  L  A  N  L  I  T  C  A  Y  A  M  V  M  M  M  V  V  I  G  I  M  L  Q
2965  ATCATGGAGGACGGTTGGCTCGCGCCGTCCAGCATGTTCACAATCATAACGTTCGGAACGTTCTTCATCACAGGCGCC
989    I  M  E  D  G  W  L  A  P  S  S  M  F  T  I  I  T  F  G  T  F  F  I  T  G  A
3043  TTGCACCCGCAGGAAATAAACTGCCTGTTGTACTTGGGCATCTACTTCATTACCATTCCGAGTATGTACATGTTGCTG
1015   L  H  P  Q  E  I  N  C  L  L  Y  L  G  I  Y  F  I  T  I  P  S  M  Y  M  L  L
3121  ATCATTTACTCGCTGTGCAATCTCAACAACGTGGCGTGGGGCACGAGGGAGGTGGCGGTGAAAAAGACTGCTAAGGAT
1041   I  I  Y  S  L  C  N  L  N  N  V  A  W  G  T  R  E  V  A  V  K  K  T  A  K  D
3199  ATGGAAATGGAGAAGAAAGCGGCCGAGGAAGCGAAGAAGGCCATGGACAACAAGAGTATGTTGCGGTGGTTCGGCAAC
1067   M  E  M  E  K  K  A  A  E  E  A  K  K  A  M  D  N  K  S  M  L  R  W  F  G  N
3277  GACAAGTCCGACGATAAGAGCGGCTCCATGGAGGTCAGCGTGGCCGGCCTCTTTAAGTGCATGTGCTGCACCAATCCA
1093   D  K  S  D  D  K  S  G  S  M  E  V  S  V  A  G  L  F  K  C  M  C  C  T  N  P
3355  AAAGACCACAAGGAGGACCTGCACCTGCTCCAGATCGCTAACAGCATGGAGAAGATTGAGAAGAGGCTTGACGCACTA
1119   K  D  H  K  E  D  L  H  L  L  Q  I  A  N  S  M  E  K  I  E  K  R  L  D  A  L
3433  GGTGCTAAAACGGAGGTAGATGAAAAGCCACCTTCGCGCCGGCGCTCTTCCATGGGCCTGCGCAGCGACACCCTGTCC
1145   G  A  K  T  E  V  D  E  K  P  P  S  R  R  R  S  S  M  G  L  R  S  D  T  L  S
3511  ATGCTGCCCGAGTACGAAGACAGCGAAGTCTCCACAGAAATACCTCGAGAAGAACGCGACGACCTAATCAATCCGTTC
1171   M  L  P  E  Y  E  D  S  E  V  S  T  E  I  P  R  E  E  R  D  D  L  I  N  P  F
3589  TGGATCGAAGATCCCAGCATACAGAAAGGCGAAGTCGACTTCCTGACCACGGCCGAAACAAATTTCTGGAAAGACATG
1197   W  I  E  D  P  S  I  Q  K  G  E  V  D  F  L  T  T  A  E  T  N  F  W  K  D  M
3667  ATCGACACATATTTGAGACCGATTGATGAAAACAAGGAGAAACAGGAACGTATCAAAGCAGACTTGAAGGCGCTACGC
1223   I  D  T  Y  L  R  P  I  D  E  N  K  E  K  Q  E  R  I  K  A  D  L  K  A  L  R
3745  GACAAGATGGTCTTTGCTTTTCTGATGCTGAATGCGCTCTTCGTGGTCGTCATCTTCCTGCTGCAGCTCAACCAGGAC
1249   D  K  M  V  F  A  F  L  M  L  N  A  L  F  V  V  V  I  F  L  L  Q  L  N  Q  D
3823  CAGCTGCACTTGCAGTGGCCGTTTGGACAGAAGGTTCAGATAATTTACGACGGGGACGCAAATTTGGTGATCATCGAC
1275   Q  L  H  L  Q  W  P  F  G  Q  K  V  Q  I  I  Y  D  G  D  A  N  L  V  I  I  D
3901  CGCGAGTACCTGACGCTGGAGCCGATCGGGTTTCTGTTCGTGATCTTCTTCGGGTTCGTTATCATTATCCAGTTCACC
1301   R  E  Y  L  T  L  E  P  I  G  F  L  F  V  I  F  F  G  F  V  I  I  I  Q  F  T
3979  GCCATGCTGTTCCATCGCTTCGGCACGGTCGTGCACTTGCTGGCTAATACACAACTCAACTGGTATTGCACTCGAAAG
1327   A  M  L  F  H  R  F  G  T  V  V  H  L  L  A  N  T  Q  L  N  W  Y  C  T  R  K
4057  CCAGACGAGTTGACGCAACACGCTCACATCGAGAAGAACGCGATCGAGATAGCGAAGGACCTGCAGAAGCTGAACACT
1353   P  D  E  L  T  Q  H  A  H  I  E  K  N  A  I  E  I  A  K  D  L  Q  K  L  N  T
4135  GACGACGTGGACCGCGCCGGCGTCGACCAGGCGCCGGTGACGCGGCGTCGCACGCTGCACAACCTGGAGCGCGCGCGG
1379   D  D  V  D  R  A  G  V  D  Q  A  P  V  T  R  R  R  T  L  H  N  L  E  R  A  R
4213  GAGACCAAGCACAGCACGACCAACCTGGACGCCAACTTCAAGCGCCGTCTTATGAACCCCGAGACTGACATAATAGCG
1405   E  T  K  H  S  T  T  N  L  D  A  N  F  K  R  R  L  M  N  P  E  T  D  I  I  A
4291  CGCATGTCGTCGCTGGGCGGCAACACGGCGATGCGTCGCGCGACGCTGCGCGCGCTGCAGACGCGGCGCGAGTCGGTG
1431   R  M  S  S  L  G  G  N  T  A  M  R  R  A  T  L  R  A  L  Q  T  R  R  E  S  V
4369  CTGGCGGAGCGGCGCCAGTCGCAGCTGCAGCAGGCGCGCGGCTCCACCACTGAGTTCCTCTACGCTGCGCCCAGTCCT
1457   L  A  E  R  R  Q  S  Q  L  Q  Q  A  R  G  S  T  T  E  F  L  Y  A  A  P  S  P
4447  GCCGGCAATATGGGTGACCGCGTTGGGCGTCGCATAACTGGCGTGTACGCCAACAAGGGCTACGAGCCGGCGCTGGAC
1483   A  G  N  M  G  D  R  V  G  R  R  I  T  G  V  Y  A  N  K  G  Y  E  P  A  L  D
4525  AGCGACGAGGAGGACACCCCGCGCCCGCGCCGCAACACCGTACGATTCCAAAATAGCAACTTCTGA
1509   S  D  E  E  D  T  P  R  P  R  R  N  T  V  R  F  Q  N  S  N  F  *
```

Figure S1. Nucleotide and deduced amino acid sequences of GpCHSA (A) and GpCHSB (B) of *G. pyloalis*. The putative transmembrane segments predicted by TMHMM Server v. 2.0 are in shaded gray. The potential *N*-glycosylation sites predicted by PROSCAN are marked with bold lines. The amino acid sequence of the putative catalytic domain is in black background. The signature sequences (EDR and QRRRW) involved in catalytic function are in red boxes.
